# Data Science of Stroke Imaging and Enlightenment of the Penumbra

**DOI:** 10.3389/fneur.2015.00008

**Published:** 2015-03-05

**Authors:** Fabien Scalzo, May Nour, David S. Liebeskind

**Affiliations:** ^1^Department of Neurology, Neurovascular Imaging Research Core, University of California Los Angeles, Los Angeles, CA, USA

**Keywords:** acute stroke, cerebral ischemia, neurology, neuroimaging, MRI imaging, CT imaging

## Abstract

Imaging protocols of acute ischemic stroke continue to hold significant uncertainties regarding patient selection for reperfusion therapy with thrombolysis and mechanical thrombectomy. Given that patient inclusion criteria can easily introduce biases that may be unaccounted for, the reproducibility and reliability of the patient screening method is of utmost importance in clinical trial design. The optimal imaging screening protocol for selection in targeted populations remains uncertain. Acute neuroimaging provides a snapshot in time of the brain parenchyma and vasculature. By identifying the at-risk but still viable penumbral tissue, imaging can help estimate the potential benefit of a reperfusion therapy in these patients. This paper provides a perspective about the assessment of the penumbral tissue in the context of acute stroke and reviews several neuroimaging models that have recently been developed to assess the penumbra in a more reliable fashion. The complexity and variability of imaging features and techniques used in stroke will ultimately require advanced data driven software tools to provide quantitative measures of risk/benefit of recanalization therapy and help aid in making the most favorable clinical decisions.

## Introduction

Stroke is a leading cause of death and a major cause of long-term disabilities across the globe. In the United States, 795,000 people are affected by strokes each year resulting in about $74 billion of total annual costs (1). Encouraging results of recent clinical trials [such as EXTEND-IA (2), ESCAPE (3), SWIFT-PRIME (4), and MR CLEAN (5)] have demonstrated the promise of endovascular therapies, yet we are far from a cure for stroke and much work remains to further increase the potential recovery in targeted populations. Neurovascular disorders are now recognized as a prominent public health issue worldwide due to aging populations and the socioeconomic burden of stroke. Improved utilization of stroke imaging to derive maximal knowledge from embedded data and associated underlying features may be leveraged to tailor therapeutic decisions and optimize outcomes after acute stroke.

In the best-case scenario, the treatment of acute ischemic stroke can lead to a full recovery. This generally happens when revascularization occurs early enough. If revascularization is not achieved or if it occurs too late, chances of recovery decrease and severe complications associated with devastating neurological effects become more likely. Revascularization, or rapidly reopening occluded arteries, is therefore the main interventional strategy in acute ischemic stroke. The purpose of such therapy is to restore perfusion in the ischemic tissue. As of today, the only FDA-approved therapy for reopening vessels in acute ischemic stroke remains intravenous tissue plasminogen activator (tPA) (6). Endovascular interventions such as intra-arterial thrombolysis and mechanical thrombectomy have both demonstrated potential for improved outcomes after stroke. The possible benefits of these therapies have to be carefully balanced with the concomitant risks, including hemorrhagic transformation (7) and other complications (8). The most common way to estimate the potential benefit is by measuring the extent of the salvageable ischemic tissue, previously described as the penumbra.

This paper provides a perspective on the penumbra by identifying a few challenges that render the characterization and definitions uneasy and by discussing the lessons that we have learned so far and toward where they could lead in the future of stroke care. After describing the current clinical practice (see Current Clinical Practice) and pathophysiology (see Pathophysiology of Cerebral Ischemia), we review imaging based definition of the penumbra. We then describe the heterogeneity of acute stroke in terms of imaging patterns (see Imaging Definitions of Penumbra). Finally, we discuss in Section “MRI and CT Definitions,” how advanced methods such as computer vision and machine could help to bring a new dimension to the understanding and definition of salvageable tissue.

## Current Clinical Practice

Cerebral ischemia is a dynamic process that spans from hyperacute presentation to acute, subacute, and chronic phases. Minimizing the time elapsed from stroke onset to treatment has been a priority target of current clinical practice (9) as acting early and decisively becomes integral to improving patient outcomes.

While the safety time window for IV-tPA administration is 3 h in all and 4.5 h in a subset of patients, exact time of stroke onset is usually unknown and the onset of symptoms is used as an indirect marker of stroke. Last known well time is widely used as the standard to designate stroke onset. It is, however, an approximate variable for two main reasons. First, about 15% of all strokes occur during sleep and therefore the time of onset for those patients is only approximate. Second, the last known well time, or the start of the detectable symptoms may not correspond to the exact time of the true stroke onset. Time is a relative notion in stroke as it has been shown that the dynamics of lesion growth and the rate of cell death in the ischemic territory vary drastically from patient to patient. Several studies (10) have shown that some patients could benefit from thrombolysis and recanalization procedures after the 4.5 h window. However, variability of response and increased risk associated with late therapies leads to heterogeneous results related to successful recovery.

Facing this uncertainty, the selection of patients who may benefit from reperfusion therapy is one of the most critical tasks in acute stroke care. Evidence suggests that time alone is not sufficient to optimally select patients and that neuroimaging can play an influential role in refining treatment decisions. Imaging, such as multimodal CT, MRI, and angiography, have been increasingly used and reflect a snapshot of the state of the brain tissue at a point in time. The most important task of imaging has been to rule out intracranial hemorrhage for determination of tPA eligibility. Visual examination of non-contrast CT or GRE offer high accuracy in detecting any sign of hemorrhage (11). Beyond exclusion for safety reasons, neuroimaging is used to determine eligibility for endovascular treatments and quantification of possible benefits. This is done through identifying at-risk, but viable and ischemic tissue. The penumbra is seen as the target tissue for revascularization therapies as it is thought of as viable but tissue at risk of becoming irreversibly infarcted. When reperfusion or collateral circulation is established, these areas may recover. Without reperfusion, such brain cells in the penumbra will die, and the lesion will expand. In addition to the assessment of the penumbra, the collateral status of the involved cerebrovascular territory, which represents the quality of the blood flow diversion in the presence of arterial occlusion, is also recognized as a predictor of poor outcome. Although multimodal CT or MRI can be used to characterize acute strokes, guide treatment decisions, and evaluate recovery, the image acquisition, processing, and interpretation are complex and time-consuming and may lead to unnecessary delays in care. There is an overt need to accelerate imaging protocols and extract imaging markers to guide clinical decisions in acute stroke.

## Penumbra in Perspective

### Pathophysiology of cerebral ischemia

The ischemic penumbra is described as the cerebral parenchyma adjacent to the area of dense ischemic infarction. Early on, multiple animal models were used to examine the limits of cerebral blood flow (CBF) volumes at which cerebral ischemia has functional implications on neuronal networks (12–16). Astrup et al. (17) described the ischemic core and penumbra as a ring of parenchyma surrounding an area of dense ischemia at the center. They further described the ischemia as existing in a range between a threshold of electrical failure as in the penumbra contrasted with a threshold of energy and ion pump failure that exists in the ischemic core. The greatest value hence in defining the ischemic penumbra is perhaps the potential that exists for its salvage by timely restoration of blood flow prior to reaching the threshold for neuronal cell death. The ischemic cascade in itself involves both apoptotic and necrotic cell death mechanisms (18). Patterns of cerebral arterial and venous blood flow have been characterized as consistent with autoregulation, oligemia, ischemia, or irreversible injury by using positron emission tomography (PET) and obtaining objective information with regards to CBF, CBV, metabolic rate of oxygen, and oxygen extraction (19). However, the use of PET imaging in acute stroke remains limited due to impracticability.

### Imaging definitions of penumbra

Neuroimaging plays a major role at several stages during the clinical management of patients treated for acute ischemic stroke. It is used to confirm diagnosis of stroke with respect to symptomatic presentation and clinical examination, to determine blood flow pathology by locating the stenosed or occluded vessel, to direct treatment decisions by identifying ischemic tissue or presence of hemorrhage, to guide endovascular procedures, and to assess treatment response and neurological recovery of the patient. In this section, we review imaging definitions of the penumbra that are used to estimate the benefits of an endovascular procedure and balance them with the risk of complications.

#### MRI and CT definitions

The availability of MRI in clinical practice permits estimation of the infarct core and the extent of penumbral tissue. The infarct core is detected as the volume of abnormal diffusion-weighted image (DWI). The volumetric difference, or mismatch, observed between the DWI and perfusion-weighted image (PWI) abnormalities is considered as a reliable indicator of salvageable tissue at risk (20–22). TTP or *T*_max_ parameters, extracted from PWI images, are thresholded to obtain a volume of hypoperfused but viable tissue. While a meaningful volume of penumbral tissue supports the decision for reperfusion therapy, there is no consensus in the computation of mismatch [it is usually in the range *T*_max_ > (2, 10) s] or on the exact definition of what significant mismatch constitutes (23, 24). In a systematic study of the patients enrolled in the DEFUSE study (25), it was found that a mismatch ratio of 38% provided the highest accuracy for identifying patients in whom reperfusion was associated with a favorable response (for a *T*_max_ threshold of 2 s).

Although MR perfusion studies accurately detect early signs of ischemia, it is contraindicated for some patients (e.g., with metallic foreign body or claustrophobia), not available in many institutions, and may not be utilized in a timely fashion. CT perfusion (CTP), on the other hand, is usually associated with lower cost, greater availability, and faster imaging. It has been established as an attractive alternative imaging method in many stroke care facilities. Although MR perfusion is more sensitive to early ischemic changes, the parameter maps extracted with CTP and MR perfusion are closely correlated. CTP can delineate infarct core and penumbral tissue using CBF and CBV thresholds of 34% (in comparison to a region defined in the healthy hemisphere) and 2.5 mL/100 g, respectively (26). These thresholds allow for automatic computation of infarct core and penumbral maps comparable to the ones obtained from MR perfusion (27). With the extraction of more advanced feature maps from perfusion studies (as described in the subsequent paragraphs), it is anticipated that some features extracted from MRI may not be equivalent with CTP. Such competitivity might be beneficial to bring further imaging advances in the long run. The importance of continued focus on developing these imaging modalities and in streamlining protocols lies in the potential for preventing further neurological deterioration in patients presenting with acute ischemic stroke.

#### Alternative quantitative methods

Beyond the DWI/PWI mismatch and CTP models, other imaging modalities and quantitative models have been studied to estimate the extent of viable tissue at risk. These models are typically built by analyzing voxel intensity at onset with respect to the observed tissue fate, as measured in FLAIR images several days after intervention. Wu et al. (28) evaluated a generalized linear model (GLM) based on DWI and PWI in 14 patients. Rose et al. (29) used Gaussian models trained on multiple parameters to predict tissue outcome in 19 patients. Other studies were performed based on logistic regression (30) and ISODATA (31) applied to apparent diffusion coefficient (ADC) and CBF. The main advantage of these methods is that they do not rely on specific thresholds to make predictions and can handle noisy observations better. Most recently, Kidwell et al. (32) described a voxel-based multimodal CT and MRI models aimed to effectively define penumbral patterns.

##### Regional models

**Figure 1:**
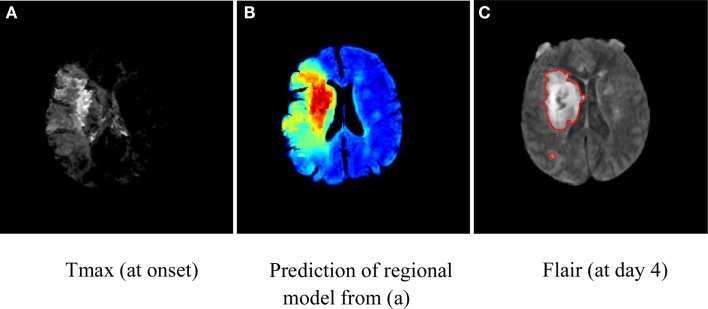
**Illustration of map of tissue outcome (B) predicted from Tmax (A) using a regional computational model**. Red areas in **(A)** depict tissue likely to be infarcted (despite intervention), while green areas represent tissue at risk and can be thought of as penumbral tissue. The groundtruth in terms of Flair (at day 4) is shown in **(C)**.

Infarct growth rate or the evolving ischemic core is quite variable, likely driven by collateral status, and may spatially vary over time due to regional hemodynamic compromise. Even in some cases of successful revascularization, the ischemic core may still expand into nearby or adjacent brain tissue. Consequently, a healthy voxel surrounded by injured tissue at early stages is more likely to become irreversibly damaged even though it may not meet the criterion to be labeled as tissue at risk. Unlike previously mentioned quantitative models that consider each voxel independently, research efforts (33) have shown that the regional distribution of intensities surrounding a voxel at early stages may capture characteristics about the dynamic of lesion growth and be predictive of tissue outcome (Figure 1). These studies have integrated regional information by exploiting spatial correlation between voxels (34), prior map of spatial frequency-of-infarct (35), and neural networks (36). Such emerging approaches offer potential refinement of single voxel-based models of penumbra.

##### Gradient: a glimpse at spatiotemporal changes

**Figure 2:**
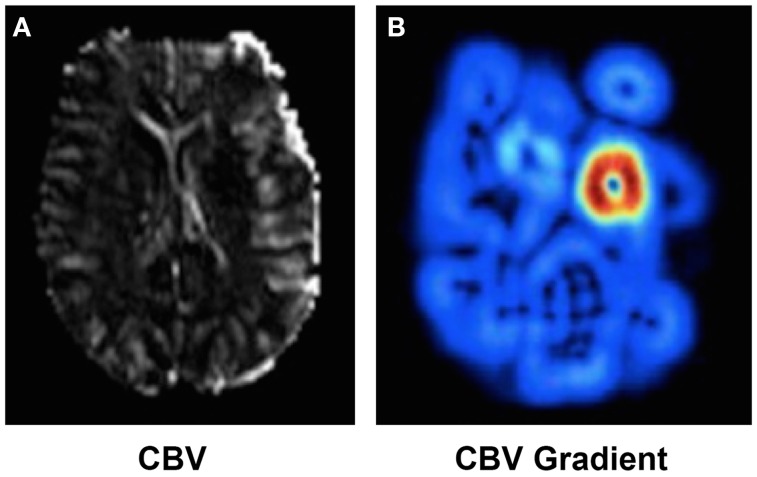
**CBV (A) and CBV (B) gradient map computed in presence of a MCA occlusion**. High CBV gradient (shown in red in **(B)**) indicates areas at risk of collateral failure.

Finding the optimal threshold of a single parameter that generalizes across a diversified patient population is not trivial and beyond current methods. Instead, recent studies (37) have indicated that the local gradient (i.e., relative spatial change) of a parameter may be a complementary predictor. Gradient images can be computed reliably using a series of image filtering operations. CBV-gradient maps were retrospectively studied on 42 acute MCAO cases with serial MRI (37). CBV is an essential measure of perfusion in acute ischemic stroke that is biphasic in nature; it exhibits peripheral hyperemia (increase) and central collapse (decrease) near the ischemic core. When used to detect ultimate infarction, CBV often underestimates final volume. CBV gradients, on the other hand, represent the propensity for hemodynamic failure to distinguish benign hyperemia from penumbra surrounding the ischemic core. As seen in Figure 2, CBV gradient maps are able to demonstrate a concentric region of abnormality around the ischemic core. CBV gradient maps are able to accurately classify voxel outcome defined as infarction on day 5 fluid attenuation inversion recovery sequences, correctly predicting voxel-based hemodynamic failure. Although CBV gradient is not observed on uniformly low distributions of CBV, it can depict zones around the ischemic core that are vulnerable to hemodynamic failure and infarct evolution; thus refining further the estimation of the penumbra.

##### Perfusion angiography

MRI-based estimates of penumbra are only feasible in the acute phase and the follow-up of treatment. If the patient is deemed eligible for endovascular thrombectomy, a significant time lapse may occur from MRI; thus leading to possible infarct growth and inaccurate penumbra volume estimates due to time inconsistencies. Estimation of viable tissue through routine biplane angiograms is currently being investigated in research settings and may become available during thrombectomy in the near future. Dedicated processing software, such as perfAngio**^®^**, can process the angiogram within seconds to extract perfusion parameters. It stems from video densitometry theory that relates blood flow to the observed image intensity. Parameters such as CBF, CBV, MTT, and TTP can be displayed in color-mapped images. While animal studies (38) have demonstrated that absolute flow could be estimated precisely from DSA, it has several practical limitations in the acute stroke setting. Challenges arise in such approaches as the diameter of the vessels and exact parameters of the x-ray imaging system are typically unknown. While raw DSA data are generally used to visualize flow at the macrovascular level (large blood vessels), flow parameters extracted with DSA perfusion provide insights about the microvascular circulation as recently demonstrated in a recent animal study (39).

Although these alternative models of the penumbra may provide more accurate raw prediction of tissue outcome in presence of reperfusion, they have yet to be effectively translated into clinical decision support tools.

### Heterogeneity of ischemic stroke and potential of stroke imaging

Generalization in stroke care is challenged by the wide variability of symptoms presentation and outcomes observed across patients. Identifying population subgroups that may share similar outcomes after stroke may be identified by specific patterns in stroke imaging. Importantly, such data-driven approaches using the examples of imaging techniques highlighted above may ultimately permit tailored therapies for the individual stroke patient. Even with revascularization of various stroke patients presenting with proximal middle cerebral artery occlusion, the heterogeneity of collateral status and patterns of ischemic injury may limit our ability to predict subsequent outcomes. Unfortunately, routine clinical parameters and even basic imaging variables used in clinical practice cannot reliably portend expected outcomes. For instance, age and stroke severity remain highly influential variables in determining stroke outcomes across a population, yet such variables may be less informative for predicting the outcome of an individual patient.

Distinct lesion patterns are known to occur depending on the severity, location, and evolution of a stroke. Significant research efforts have been devoted to study if specific lesions patterns would help prediction of early prognosis of three different time points after ischemic stroke: unstable hospital course, recurrence of stroke, and poor neurological outcome at 3 month follow-up. Bang et al. (40) classified DWI lesions into six groups: territorial, other cortical, small superficial, internal border zone, small deep, and other deep infarcts. The study focused on 426 patients with acute cerebral infarcts within the middle cerebral artery territory and any recurrent strokes and prognosis at 3 month follow-up were recorded. DWI lesion pattern was a stronger and more consistent predictor of outcome than DWI lesion volume. Such results indicate that the DWI lesion pattern may help in recognition of likely differences in the early prognostic endpoints after ischemic stroke, and DWI analysis may guide targeted interventions to prevent negative outcomes.

### Computer vision and machine learning for clinical decision support

Patient selection and clinical decisions in acute stroke are often guided by review of noisy raw images or use of complex imaging features that requires a high level of expertise and experience. For example, although collateral grade is an important predictor of outcome, it is not trivial to assess and as a result, very few neurologists routinely grade collateral status. To circumvent such issues in the complexity of stroke imaging and their visual interpretation in real time, the solution has been so far to simplify, and perhaps over-simplify, the amount of information used so that it can become readily amenable to make a decision. One can easily argue that inclusion criteria in current clinical trials have so far followed the same logic with specific cut-off mismatch volume values, etc. With the availability of big data analytics, modern computer vision techniques and machine learning algorithms are revolutionizing many aspects of daily life. Several domains (such as finance and weather forecasting) where fast and accurate decisions have to be made based on a very complex amount of information have already started to integrate machine learning in their decision-making process. The real purpose of these technologies is not to replace the clinician, but rather to provide a translation of the data into a more meaningful representation. While computer vision methods can be developed to extract subtle visual features from complex images, machine learning algorithms can be designed to combine a very large amount of complex information into a more easily interpretable “benefit” or “risk” score, with associated confidence value. The application of such methods in stroke imaging may lead to automated, objective quantification of images and would avoid issues of operator or reader dependence.

## Conclusion

Stroke imaging techniques provide extensive data on the pathophysiology of acute ischemic stroke, including the therapeutic target of the penumbra. Clinical decision-making and selection strategies for therapeutic interventions, formulated from estimates of risk–benefit associated with penumbral extent, may be enhanced with extraction of detailed features currently untapped in routine clinical practice. Data science of routine multimodal CT or MRI studies may yield clinically relevant knowledge to improve patient outcomes in the future. Such recently developed techniques will be further bolstered in coming years as increasingly larger imaging datasets may be shared from around the world. Ultimately, practical automated computer vision and machine learning approaches may provide critical information in real time, immediately prior to and even during therapeutic interventions.

## Conflict of Interest Statement

The authors declare that the research was conducted in the absence of any commercial or financial relationships that could be construed as a potential conflict of interest.
